# Hybrid Surgical–Catheter Epicardial Ablation of Ventricular Tachycardia: A Case Series

**DOI:** 10.3390/jcm15103782

**Published:** 2026-05-14

**Authors:** Alessandro Telesca, Roberto Scacciavillani, Gemma Pelargonio, Cristina Conte, Federico Ballacci, Federica Giordano, Francesco Perna, Gianluigi Bencardino, Francesco Spera, Gaetano Pinnacchio, Andrea Scapigliati, Massimo Massetti, Francesco Burzotta, Massimo Imazio, Maria Lucia Narducci

**Affiliations:** 1 Cardiothoracic Department, University Hospital Santa Maria Della Misericordia, Azienda Sanitaria Universitaria Friuli Centrale, 33100 Udine, Italy; cristinaconte93@gmail.com (C.C.);; 2 Department of Cardiovascular Sciences, Fondazione Policlinico Universitario Agostino Gemelli IRCCS, 00168 Rome, Italy; roberto.scacciavillani@gmail.com (R.S.); gaetano.pinnacchio@policlinicogemelli.it (G.P.);; 3 Department of Cardiovascular and Pulmonary Sciences, Catholic University of the Sacred Heart, 20123 Rome, Italy; 4 Azienda Ospedaliero Universitaria Sant’Andrea, 00189 Rome, Italy; 5 Intensive Care Unit, Department of Cardiovascular Sciences, Fondazione Policlinico Universitario “a. Gemelli” IRCCS, 00168 Rome, Italy; 6 Department of Medicine (DMED), University of Udine, 33100 Udine, Italy

**Keywords:** ventricular tachycardia, hybrid, epicardial ablation

## Abstract

**Background:** Epicardial mapping and ablation of ventricular tachycardia (VT) are used in different clinical situations but pericardial adhesions following prior cardiac surgery or previous epicardial procedures may limit a percutaneous approach. The objective of this case series is to evaluate the safety and feasibility of a hybrid approach with surgical epicardial access as a valid alternative when pericardial space is not accessible percutaneously. **Methods:** After a complete preprocedural evaluation, four patients with prior cardiac surgery underwent hybrid VT ablation under general anesthesia. Surgical subxiphoid access was performed in three cases and one patient was subjected to median resternotomy for concomitant open-heart surgery. Epicardial electroanatomic voltage maps were acquired using the CARTO 3 system (Biosense Webster) or NavX (St. Jude Medical) and VT ablations with irrigated catheters were performed. The procedural endpoint was VT non-inducibility and/or LAVA/LP abolition. **Results:** No serious periprocedural complications occurred after hybrid VT ablation. Three patients had no complex ventricular arrhythmias after a median follow-up of 43 months. A symptomatic sustained VT relapsed in one patient, without requiring a redo ablation procedure but responded to escalation of antiarrhythmic therapy. **Conclusions:** A carefully planned hybrid VT ablation with surgical epicardial access is a safe and feasible procedure in patients with epicardial scar-related re-entry circuits and pericardial adhesions that limit a percutaneous approach.

## 1. Introduction

A percutaneous approach for epicardial ablation of ventricular tachycardia (VT) was first described by Sosa and colleagues in the late 1990s, representing a major advancement in the interventional management of ventricular arrhythmias, and it has been subsequently widely used in numerous settings such as ischemic and non-ischemic cardiomyopathy, valvular heart disease and arrhythmogenic cardiomyopathy [1]. Over time, this technique has become an important adjunct to endocardial ablation, particularly in patients with a suspected epicardial substrate.

The main limitation of this approach is represented by pericardial adhesions, usually seen after prior cardiac surgery [2], that can significantly hinder catheter manipulation and limit adequate mapping and ablation of the arrhythmogenic substrate. Lysis of such adhesions is sometimes feasible from a percutaneous approach but the risk of serious complications is high [3,4]. Therefore, a hybrid strategy with surgical epicardial access is a valid alternative in this setting, enabling the operators to circumvent such limitations and potentially improving procedural efficacy and safety.

We assessed the safety and feasibility of hybrid VT ablation in a series of four patients treated at our institution from 2017 to 2020. The aim of this series was to provide additional insight into the clinical applicability of this strategy in a real-world setting.

## 2. Materials and Methods

### 2.1. Patient Population

This case series describes four procedures that were performed between July 2017 and April 2020 in a hybrid electrophysiological (EP) lab at the Policlinico Gemelli Hospital in Rome. All patients underwent a surgical combined epicardial access and VT ablation after failed endocardial ablation. In particular, a percutaneous epicardial approach had been attempted in three patients without success due to the presence of extensive pericardial adhesions from prior cardiac surgery. A complete preprocedural evaluation including imaging and review of previous invasive mapping data was performed in all cases. Preprocedural imaging included echocardiography and, when available, cardiac magnetic resonance to better define the arrhythmogenic substrate and pericardial anatomy. Recurrence of VT and the electrocardiographic (EKG) features suggestive of an epicardial site [5] were considered indications for VT epicardial ablation. The decision to proceed with a hybrid approach was made after careful evaluation by a dedicated heart team including electrophysiologists, anesthesiologists and cardiac surgeons. Baseline patient demographic and procedural data were extracted from electronic patient records.

### 2.2. Hybrid Procedure (Surgical Approach, Mapping and Ablation)

All procedures were performed under general anesthesia with endotracheal intubation in the hybrid EP operating room. A standardized anesthetic protocol was adopted to ensure hemodynamic stability throughout the procedure. Propofol 1 mg/kg, cisatracurium 0.15 mg/kg, and fentanyl 1 mcg/kg were used for induction while maintenance involved sevoflurane at 1%, remifentanil at 0.05 mcg/kg/min, and propofol as needed. Intraoperative monitoring included peripheral pulse oximetry (SpO_2_), end-tidal CO_2_ (EtCO_2_), 8-lead EKG, invasive arterial blood pressure via radial artery, central venous pressure with a central venous catheter in the internal jugular vein, hourly urine output, and bispectral index.

The surgical approach was individualized on the likely ablation target identified during preprocedural evaluation (EKG, imaging and previous endocardial mapping). This tailored strategy was aimed at optimizing exposure of the suspected arrhythmogenic substrate while minimizing surgical invasiveness. Median sternotomy was performed in one case for the concomitant need of cardiac surgery and a surgical subxiphoid window was obtained in three patients. Following surgical access, the pericardium was opened and lysis of adhesions was completed under direct visualization to achieve an adequate exposure of the epicardium and to introduce a sheath into the pericardial space to place a mapping catheter. Direct visualization facilitated safer dissection of adhesions and reduced the risk of inadvertent injury to cardiac structures.

In all patients an epicardial high-density electroanatomic voltage mapping during sinus rhythm or paced rhythm (cuts-off: 0.5–1.5 mV) was performed with the CARTO 3 system (Biosense Webster, Irvine, CA, USA) or the NavX system (St. Jude Medical, St. Paul, MN, USA). In one case concomitant endocardial mapping and ablation was performed with a retroaortic approach after intravenous administration of heparin (target activated clotting time of 250–350 s).

After substrate mapping, a programmed ventricular stimulation was performed from the right ventricular (RV) apex catheter to induce the clinical VT. If the VT was hemodynamically tolerated, an activation mapping and entrainment maneuvers were performed and radiofrequency (RF) deliveries were applied on the sites of diastolic potentials during ventricular arrhythmia. This approach enabled precise identification of critical isthmus sites sustaining the tachycardia. If VT was not hemodynamically tolerated, substrate ablation was attempted with the endpoint of scar homogenization and elimination of all areas of local abnormal ventricular activities (LAVAs) and late potentials (LPs). Using irrigated ablation catheters (SmartTouch SF for Biosense Webster or FlexAbility for St. Jude Medical) epicardial-only (or endo-epi) ablation was performed with RF power (power range: 30–45 W). Power titration was adjusted according to local electrogram characteristics and proximity to critical structures. The procedure was considered successfully closed when a complete abolition of LAVAs/LPs was achieved and non-inducibility of VT was proven post-RF with a complete stimulation protocol.

At the end of the procedure the pericardial sheath was removed and a pericardial drain was left after surgical closure until next day. Patients were successfully awakened, extubated and transferred to the intensive care unit (ICU) to be monitored for the initial 24 postoperative hours. Postoperative management focused on early detection of complications such as bleeding, pericardial effusion, or arrhythmia recurrence.

## 3. Results

### 3.1. Patient Population

We performed hybrid VT ablation in four male patients with ischemic cardiomyopathy (ICM) and a mean left ventricular ejection fraction (LVEF) of 38.2 ± 3.1% (32–42%). The mean age was 69 ± 6 years (64–82 years). Three patients were implantable cardioverter defibrillator (ICD) carriers (*pt 1*, *pt 2*, *pt 3*) and suffered from symptomatic VTs requiring multiple ICD therapy episodes despite pharmacological treatment with different classes of antiarrhythmic drugs (beta-blockers, amiodarone and mexiletine). These patients had a history of endocardial VT ablations (two in *pt 1* and *pt 2*, one in *pt 3*) and failed percutaneous epicardial approach attempts due to extensive pericardial adhesions following prior cardiac surgery. All patients had undergone coronary artery bypass graft (CABG) surgery, with associated mitral valve (MV) repair in two of them (*pt.2*, *pt.3*). One patient (*pt.4*) underwent a concomitant endo-epicardial ablation seven days after CABG for symptomatic monomorphic VTs which were persistent despite surgical revascularization. All patients were evaluated with invasive coronary angiography to confirm the patency of the grafts and to assess the anatomic proximity to targeted regions of ablation.

Baseline patient demographic data are described in Table 1.

### 3.2. Hybrid Procedure (Surgical Approach, Mapping and Ablation)

Surgical access to the pericardium was successful in all patients. In three of them (*pt 1*, *pt 2*, *pt 4*) a surgical epicardial approach with a subxiphoid window was preferred, while in *pt 3* epicardial mapping and ablation were performed with a median resternotomy justified by a concomitant planned cardiac surgery (MV replacement after degeneration of preceding MV repair). Severe adhesions were found in all cases in the diaphragmatic portion of pericardium and the anterolateral portion of the LV and were the reason for failure of epicardial percutaneous access in three patients (*pt 1*, *pt 2*, *pt 4*). Mean duration time from the surgical incision to the epicardial access was 43 ± 5 min (37–51 min). Electroanatomic mapping was acquired with the CARTO 3 system (Biosense Webster) in two patients (*pt 1*, *pt 2*) and with the NavX system (St. Jude Medical) in the others (*pt 3*, *pt 4*).

**Figure 1:**
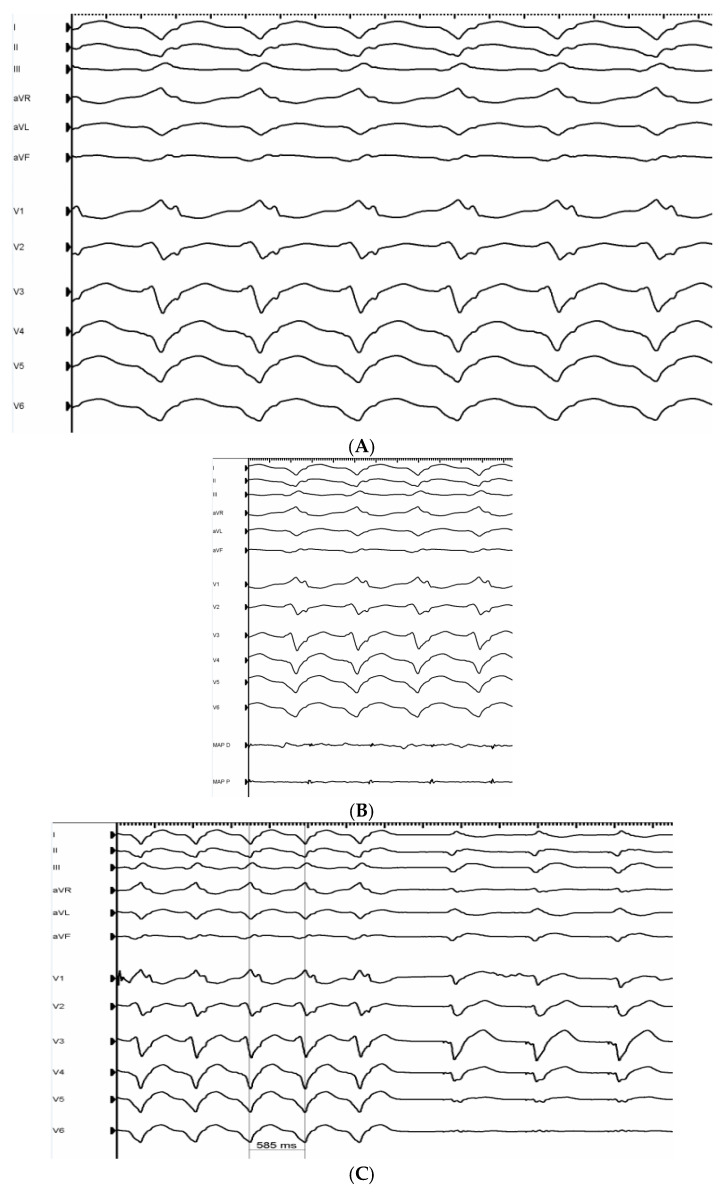

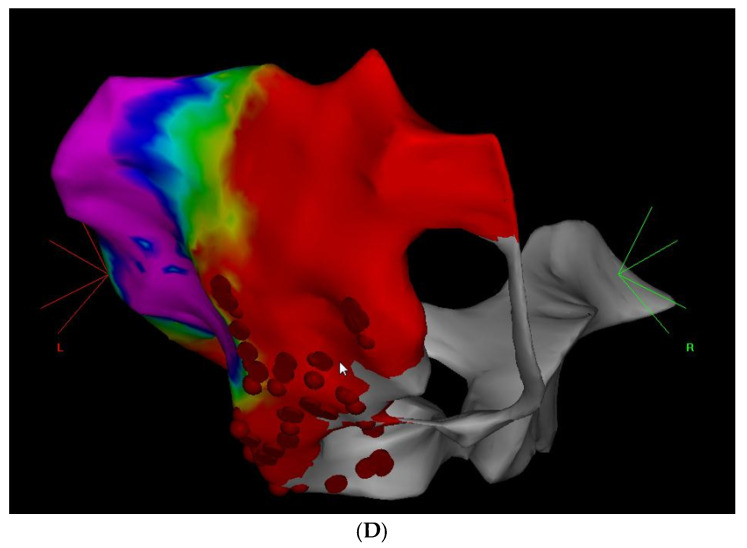
(**A**): 12-lead ECG of clinical monomorphic VT: CL of 536 ms (112 bpm), right bundle branch block morphology, QS in V4–V6 and in DI-aVL, pseudo-delta wave, wide QRS (251 ms), and MDI of 0.55. These features suggest epicardial origin of VT (VT = ventricular tachycardia CL = cycle length, MDI = maximum deflection index). (**B**): Proximal and distal electrodes of mapping catheter recording LPs during clinical VT (LPs = late potentials, VT = ventricular tachycardia). (**C**): Abrupt termination of clinical VT after slowdown of CL during RF application in inferior basal LV (VT = ventricular tachycardia, CL = cycle length, RF = radiofrequency LV = left ventricle). (**D**): PA view of the bipolar epicardial voltage map showing scar area (<0.5 mV) in the inferolateral basal and medio ventricular segments. Red dots = RF applications. (PA = postero-anterior, RF = radiofrequency).

*Patient 1* had a history of two previous endocardial VT ablations for symptomatic monomorphic VTs and recurrent ICD shock therapies. During the last endocardial approach, substrate modification at the septal and inferior border of the scar (medio-basal inferior LV wall) was performed with complete abolition of late potentials (LPs). During the activation mapping of clinical hemodynamically stable VT induced with programmed stimulation protocol, presystolic or mesodiastolic endocardial potentials were not found. Failure of endocardial ablation to slow down the VT cycle length and to stop clinical VT, the absence of an early endocardial activation area and ECG morphology suspicious for epicardial origin of VT (Figure 1A) made us opt for an epicardial approach but the subxiphoid percutaneous access attempt failed due to dense adhesions. Hence, surgical epicardial access with a subxiphoid window was planned. The LV epicardial voltage map during paced rhythm showed low-voltage areas (<0.5 mV) in the inferolateral basal and medio ventricular segments (Figure 1D). Presystolic potentials were found during activation mapping in the inferior basal LV, which was the target of multiple successful RF deliveries (resulting in termination and non-inducibility of clinical VT). Abolition of LPs in the inferolateral basal segment was also performed (Figure 1B,C).

**Figure 2:**
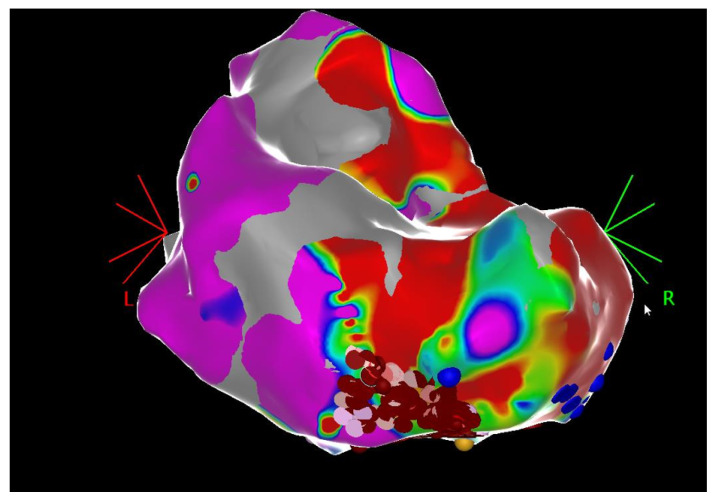
PA view of the bipolar epicardial voltage map showing low-voltage areas (<0.5 mV) in the inferior basal segments. Red dots = RF applications. (PA = postero-anterior, RF = radiofrequency).

*Patient 2* suffered from many episodes of unstable fast VT (cycle length 240 ms) interrupted by ICD shocks. Two previous endocardial ablations were performed in the LP sites (LV inferior and posteroseptal areas) without clinical success. Since the failure of percutaneous epicardial access related to the previous history of cardiac surgery (CABG and MV repair) and subsequent pericardial adhesions, a hybrid epicardial voltage mapping with subxiphoid access was realized with RF ablation at the area of LAVAs (inferior basal segment) until complete abolition of LAVAs and LPs (Figure 2). During initial applications of RF, a hemodynamically non-tolerated fast VT was induced, promptly cardioverted with direct current (DC) shock. Hence activation mapping was not possible.

**Figure 3:**
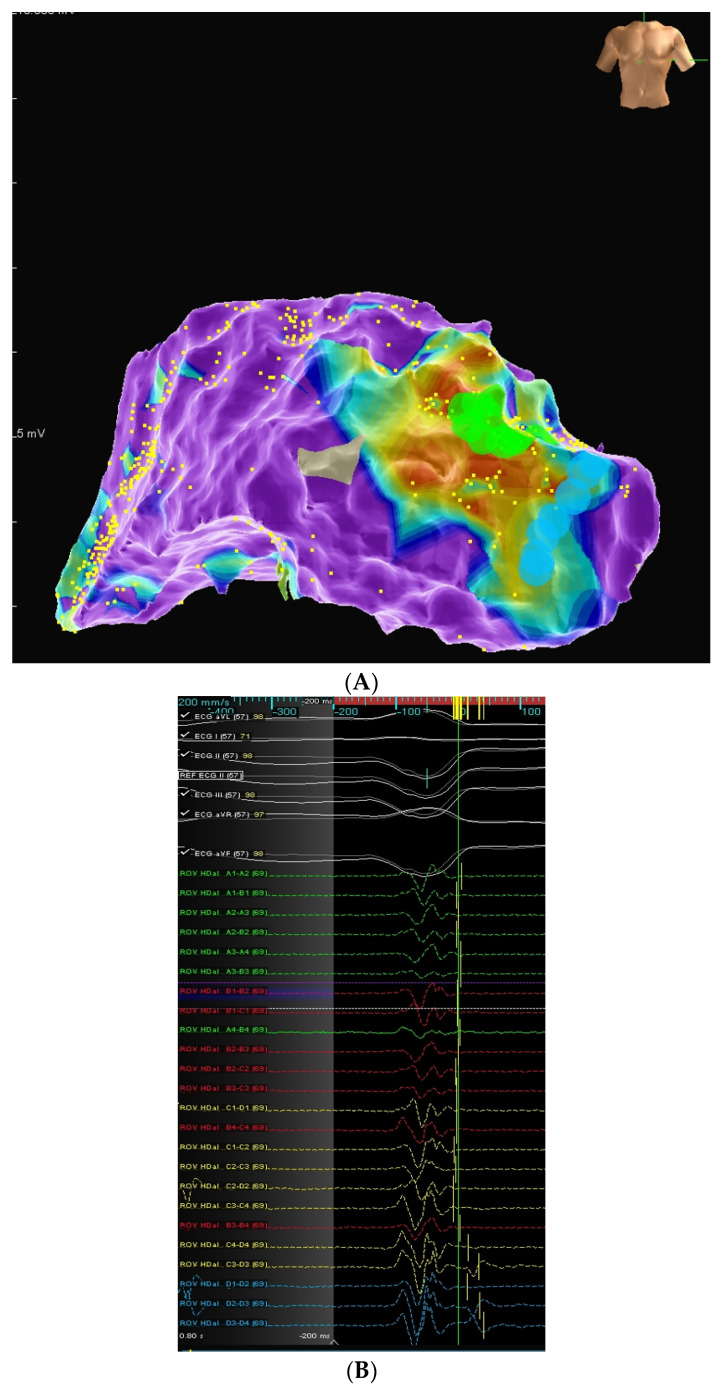
(**A**): AP view of the bipolar epicardial voltage map showing low-voltage areas (<0.5 mV) in the anterior perimitral region (AP = antero-posterior). (**B**): Mapping catheter (Advisor HD Grid, St. Jude Medical) recording local abnormal ventricular activation (LAVA) during sinus rhythm.

In *patient 3* epicardial mapping and ablation were performed with median sternotomy during a concomitant MV replacement procedure. He had undergone a previous unsuccessful endocardial VT ablation. The epicardial voltage map correlated with the endocardial one and LAVA areas were safely ablated in the anterior perimitral segment under direct visualization in a triangle between the left anterior descending coronary artery, the mitral anulus and the internal mammary graft (Figure 3A,B). Clinical VT was not inducible post-RF.

**Figure 4:**
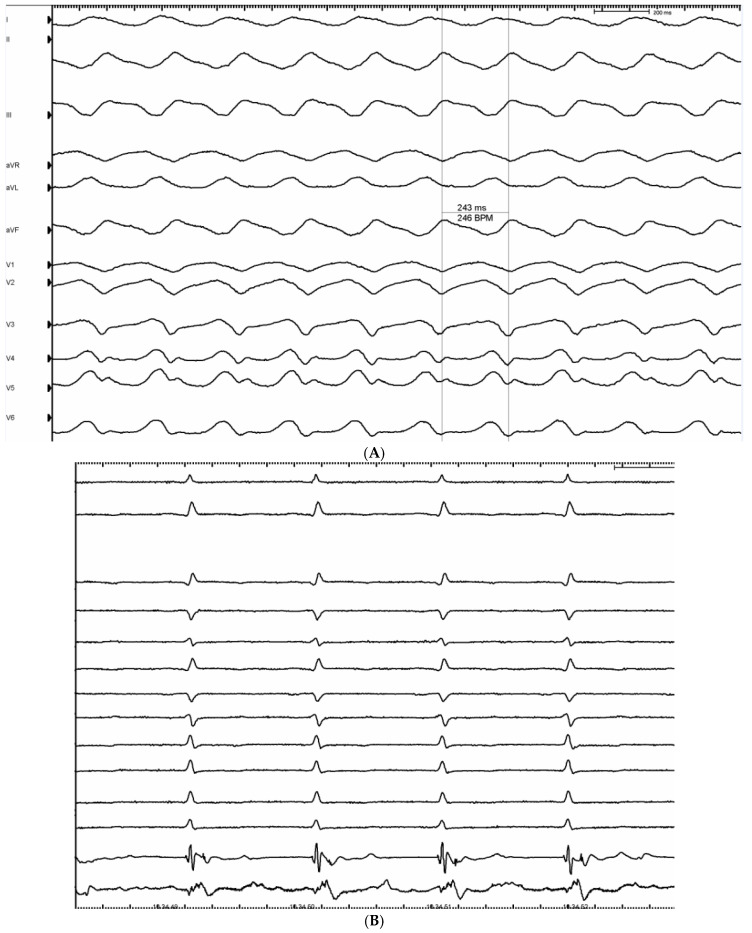
(**A**): 12-lead ECG of clinical monomorphic VT with CL of 243 ms, corresponding to 246 bpm (VT = ventricular tachycardia, CL = cycle length). (**B**): Ablation catheter (Flexibility, St. Jude Medical) recording LPs during sinus rhythm (LPs = late potentials).

*Patient 4* was admitted to the emergency room of our institution due to chest pain and palpitations with the diagnosis of symptomatic monomorphic VTs. He had three coronary stents previously implanted. A new coronary angiography showed multivessel coronary disease with indication for CABG surgery. For the persistence of monomorphic VTs despite surgical revascularization and ECG features strongly suggestive of an epicardial circuit, a hybrid surgical approach was performed after the failure of a percutaneous one (Figure 4A). LP sites in the ischemic inferior and inferolateral segments present during epicardial electroanatomical mapping were completely abolished (Figure 4B). After epicardial ablation and sternal closure, an additional LV endocardial substrate mapping and ablation with a retrograde aortic approach were performed with abolition of LAVA areas in the inferolateral and posterolateral medio ventricular endocardial LV segments. At the end of the procedure clinical VT was still not inducible, but an aggressive programmed stimulation with up to three ventricular extrastimuli induced aspecific ventricular flutter (cycle length 225 ms). An ICD was implanted in the following days.

The mean number of RF application points in our patients was 58 ± 26 (range: 21–112) and the mean ablation time was 62 ± 22 min (range: 32–94 min). The mean fluoroscopy time was 30.5 ± 10 min (range 15–49 min) while the mean total procedural time was 303 ± 73 min (range 195–443 min).

Procedural data are summarized in Table 2a,b.

### 3.3. Complications and Follow-Up

No procedure-related complications occurred in any patient except for a right groin hematoma in *patient 4* that did not require vascular intervention or blood transfusions. Transient postcardiotomy chest pain was present in all patients and successfully treated with paracetamol or non-steroidal anti-inflammatory drugs for a few days. No anesthesia-related complications were observed. Three patients (*pt 1*, *pt 2*, *pt 4*) were invasively monitored in ICU after the procedure for 1 day and then were transferred to the general ward. Only one patient (*pt 3*) was observed in ICU for 3 days due to the need for inotropic pharmacological support after median sternotomy and MV replacement. The pericardial drain was removed in all subjects the day following the ablation in the absence of active drainage or pericardial effusion at echocardiographic control. All patients were discharged from the regular ward after 7–9 days, except for *patient 1* who required a longer hospitalization (30 days) due to nosocomial pneumonia treated with endovenous antibiotic therapy. *Patient 3* was admitted to a postoperative cardiac rehabilitation unit after dischargement.

During a median follow-up period of 1171 days (IQR 691 days) only one patient died (*pt 1*) due to heart failure exacerbated by sepsis 29 months after hybrid ablation. Three patients (*pt 1*, *pt 2*, *pt 3*) were VT-free under previously ineffective antiarrhythmic therapy during follow-up, while *patient 4* presented an episode of symptomatic slow VT seven months after ablation and required introduction of amiodarone with no further arrhythmic events.

Outcomes data are described in Table 3.

## 4. Discussion

We have described the cases of four patients who underwent surgical VT ablation. This small series represents a real-world experience in a highly selected and challenging patient population. Although ventricular tachycardia in ischemic cardiomyopathy is classically associated with a predominantly endocardial substrate, a subset of patients may exhibit a significant epicardial component, particularly in the presence of prior infarction involving the inferolateral wall or in cases of prior surgical interventions altering myocardial architecture. In three cases epicardial access was attempted but resulted in failure due to previous cardiac surgery; consequently, a surgical subxiphoid window was performed. In one case the epicardial procedure was achieved with median sternotomy in the context of a planned open-heart surgery procedure, thus avoiding the risks of a percutaneous approach. Overall, these scenarios highlight the need for alternative strategies when standard percutaneous techniques are not feasible.

Several reports demonstrated a high failure rate (nearly 85%) of the percutaneous epicardial approach in patients who had undergone a previous pericardiotomy for cardiac surgery, and, in successful cases, complications rates of up to 22% due to the inherent risks of adhesion lysis, such as bleeding and coronary artery injury [4,6]. These findings underscore the technical complexity and potential hazards associated with repeat pericardial access in this subset of patients.

Surgical access to the epicardial region is a reasonable option in these challenging VT ablation cases but techniques and outcomes of this approach were addressed in a limited number of studies.

In 2004 Soejima et al. demonstrated that a surgical subxiphoid epicardial strategy was feasible for six patients with difficult percutaneous pericardial access requiring ablation of epicardial arrhythmia foci, with good outcomes and in absence of complications apart from chest pain of an inflammatory nature (reactive pericarditis) [7]. In 2010, Sacher and co-workers reported that surgical ablation was deemed necessary and performed in 14/136 patients undergoing epicardial VT ablation due to previous failed approaches, without significant complications [8]. Michowitz et al. described 14 patients with either surgical access with a subxiphoid window (11) or limited anterior thoracotomy (3) depending on the target area, in absence of major complications [9]. These studies collectively suggest that surgical access can be performed with an acceptable safety profile when carried out in experienced centers.

In a multicenter study of 444 VT ablation procedures, Sarkozy et al. documented the use of the surgical technique in 13 out of 56 epicardial procedures, with a good safety profile [10]. More recently, Li et al. found no statistical difference in long-term outcomes between subxiphoidal and thoracotomy approaches in 38 patients compared with a propensity-matched percutaneous epicardial cohort [11]. These data suggest that surgical access does not compromise long-term efficacy when compared to conventional strategies. Lately, Vroomen and colleagues published a study involving five patients who underwent hybrid ablation for recurrent sustained VT using combined endocardial and epicardial mapping and radiofrequency ablation, with no periprocedural complications and two recurrences at follow-up, with one requiring a redo endocardial procedure and the other an escalation of anti-arrhythmic drugs [12].

These studies and our data highlight not only the safety and feasibility of the hybrid ablation technique, but also its strength and weaknesses. Direct visualization of the heart and coronary arteries, in fact, decreases the risk of coronary artery and phrenic nerve damage and helps the operator reach ideal contact force and direction with the ablation catheter. Moreover, the presence of the cardiac surgeon aids in removing epicardial fat pads and in managing potential complications. This multidisciplinary approach represents a key advantage of hybrid procedures. On the other hand, the main drawbacks are represented by the increased duration of the procedure, the need for a dedicated procedural environment (such as a hybrid operating room) that limits the availability to tertiary centers only and the increased recovery time of the patient [12].

Although hybrid surgical ablation is proving to be a valuable tool for the treatment of VTs not responsive to endocardial approach, other novel techniques are emerging with good long-term outcomes in this context, such as transcoronary or retrograde venous ethanol ablation [13], needle-based catheter ablation [14] and radiotherapy ablation (STAR) [15]. The latter one seems the more promising since it is minimally invasive and also effective in older patients with many comorbidities, demonstrating good efficacy and safety profiles. Thus, radiotherapy ablation is currently seen not only as a last resort therapy in VT ablation patients but also as an alternative to conventional methods when these are not feasible, ineffective or contraindicated [16].

### Limitations

Our study carries obvious limitations related to the small number of subjects and to a single center’s experience.

## 5. Conclusions

In conclusion, our case series suggests that a carefully planned endo-epicardial hybrid VT ablation may yield good success rates in selected patients with an acceptable safety profile. This approach appears particularly useful in patients with prior cardiac surgery and failed percutaneous epicardial access. In the next future, this technique, together with developing alternatives, will represent another useful tool for challenging ventricular arrhythmias. Further larger, prospective studies are warranted to confirm these preliminary observations and to better define its role in clinical practice.

## Figures and Tables

**Table 1:** Baseline demographic data (M = male, ICM = ischemic cardiomyopathy, LVEF = left ventricular ejection fraction, CABG = coronary artery bypass graft, MV = mitral valve, VT = ventricular tachycardia, ICD = implantable cardioverter defibrillator, CRT-D = cardiac resynchronization therapy defibrillator).

Patient	Etiology	LVEF (%)/NYHA	Prior Cardiac Surgery	Reason for Ablation	Nr. of Clinical VT	Nr. of Previous Endocardial Ablation	Device	AAD on Admission
1: M 82 y.o.	ICM	32/III	CABG (2004)	Sustained VT	1	2 (2008–2017)	ICD	Beta-blockers, mexiletine
2: M 65 y.o.	ICM	40/II	CABG+MV repair (2002)	Sustained VT	1	2 (2017)	CRT-D	Beta-blockers, amiodarone
3: M 66 y.o.	ICM	39/III	CABG+MV repair (2011)	Sustained VT	1	1 (2019)	CRT-D	Beta-blockers, amiodarone, mexiletine
4: M 64 y.o.	ICM	42/II	CABG (2020)	Sustained VT	1	None	None	Beta-blockers

**Table 3:** Outcomes data (M = male, VT = ventricular tachycardia, VFl = ventricular flutter, ICU = intensive care unit, HF = heart failure).

Patient	Acute Result	Acute Complications	Nr. of Days in ICU	Nr. of Days from Ablation to Discharge	Recurrence	Death (Cause)	Follow-Up (Days)
1: M 82 y.o.	No VT	None	1	30	no	Yes (HF)	884
2: M 65 y.o.	No VT	None	1	7	no	no	1951
3: M 66 y.o.	No VT	None	3	8	no	no	1329
4: M 64 y.o.	VFl inducible, not clinical	Right groin hematoma with conservative management	1	9	Yes (1 slow VT episode)	no	1014

**Table 2:** (**a**): Procedural data (M = male, EI = endotracheal intubation, HD = high density). (**b**): Procedural data (M = male, RF = radiofrequency).

(a)						
Patient	General Anesthesia and EI	Type of Epicardial Access	Time to Access (min)	HD Electroanatomic Mapping System	Mapping Catheter	Epicardial Scar Location
1: M 82 y.o.	yes	Subxiphoid	37	CARTO (Biosense Webster)	SmartTouch SF	inferolateral basal and medio ventricular
2: M 65 y.o.	yes	Subxiphoid	45	CARTO (Biosense Webster)	SmartTouch SF	inferior basal
3: M 66 y.o.	yes	Median sternotomy	51	NavX (St. Jude Medical)	Advisor HD Grid	anterior perimitral
4: M 64 y.o.	yes	Subxiphoid	39	NavX (St. Jude Medical)	Advisor HD Grid	inferior and inferolateral
**(b)**						
**Patient**	**Ablation Set**	**Maximum Ablation Power (W)**	**Nr. of RF Applications**	**Ablation Time (min)**	**Fluoroscopy Time (min)**	**Total Procedural Time (min)**
1: M 82 y.o.	Epi only	30	56	74	31.6	266
2: M 65 y.o.	Epi only	35	44	48	25.3	195
3: M 66 y.o.	Epi only	35	21	32	15.5	443
4: M 64 y.o.	Epi-Endo	45	112	94	49.8	311

## Data Availability

The data presented in this study are available on request from the corresponding author.
